# Клинические проявления, принципы диагностики и лечения фосфатурических мезенхимальных опухолей, секретирующих фактор роста фибробластов 23: результаты наблюдения 40 случаев

**DOI:** 10.14341/probl13221

**Published:** 2023-11-10

**Authors:** С. А. Гронская, Ж. Е. Белая, Л. Я. Рожинская, Г. А. Мельниченко, Т. А. Дубовицкая, Е. О. Мамедова, С. С. Родионова, Ю. В. Буклемишев, Е. А. Пигарова, М. В. Дегтярев, Д. М. Бабаева, В. П. Владимирова, Н. В. Тарбаева, С. С. Серженко, А. Ю. Григорьев, Л. К. Дзеранова, В. Ю. Карпенко, А. Л. Карасев, Р. Н. Федотов, И. Н. Ульянова, Н. В. Торопцова, О. М. Лесняк, Н. Г. Мокрышева, И. И. Дедов

**Affiliations:** Национальный медицинский исследовательский центр эндокринологии; Национальный медицинский исследовательский центр эндокринологии; Национальный медицинский исследовательский центр эндокринологии; Национальный медицинский исследовательский центр эндокринологии; Национальный медицинский исследовательский центр эндокринологии; Национальный медицинский исследовательский центр эндокринологии; Национальный медицинский исследовательский центр травматологии и ортопедии им. Н.Н. Приорова; Национальный медицинский исследовательский центр травматологии и ортопедии им. Н.Н. Приорова; Национальный медицинский исследовательский центр эндокринологии; Национальный медицинский исследовательский центр эндокринологии; Национальный медицинский исследовательский центр эндокринологии; Национальный медицинский исследовательский центр эндокринологии; Национальный медицинский исследовательский центр эндокринологии; Национальный медицинский исследовательский центр эндокринологии; Национальный медицинский исследовательский центр эндокринологии; Национальный медицинский исследовательский центр эндокринологии; Национальный медицинский исследовательский центр травматологии и ортопедии им. Н.Н. Приорова; Национальный медицинский исследовательский центр травматологии и ортопедии им. Н.Н. Приорова; Московский государственный медико-стоматологический университет им. А.И. Евдокимова; Национальный медицинский исследовательский центр эндокринологии; Научно-исследовательский институт ревматологии им. В.А. Насоновой; Северо-Западный государственный медицинский университет им. И.И. Мечникова; Национальный медицинский исследовательский центр эндокринологии; Национальный медицинский исследовательский центр эндокринологии

**Keywords:** гипофосфатемия, фосфор, остеомаляция, опухоль, ФРФ23, остеопороз, паратиреоидный гормон (ПТГ)

## Abstract

**Обоснование:**

Обоснование. Опухоль-индуцированная остеомаляция — это редкое приобретенное заболевание, проявляющееся гипофосфатемической остеомаляцией, связанной с избыточной секрецией фактора роста фибробластов 23 (ФРФ23). ФРФ23 в норме секретируется костной тканью (остеоцитами) и регулирует обмен фосфора, способствуя его выведению почками, то есть является неклассическим гормоном.

**Цель:**

Цель. Представление клинического опыта по диагностике, лечению и реабилитации пациентов с опухоль-индуцированной остеомаляцией.

**Материалы и методы:**

Материалы и методы. В наблюдение были включены 40 пациентов с клинически диагностированной опухоль-индуцированной остеомаляцией, у 34 из которых опухоль была локализована, 27 прооперированы и 21 достигли стойкой ремиссии.

**Результаты:**

Результаты. Медиана возраста составила 48 [41; 63] лет на момент диагностики, 43% пациентов — мужчины, время от первых симптомов до установления диагноза составило 8 [4; 10] лет. Лабораторно у пациентов отмечались гипофосфатемия — 0,47 [0,4; 0,53] ммоль/л, снижение индекса реабсорбции фосфатов — 62 [52; 67]% и повышение активности щелочной фосфатазы — 183 [112; 294] Ед/л. На момент установления диагноза все пациенты имели множественные патологические переломы, передвигаться полностью самостоятельно могли лишь 10%, при этом все испытывали болевой синдром, в том числе 77,5% больных охарактеризовали боль выше 8 баллов (по 10-балльной шкале). Среди методов, используемых для обнаружения опухолей, самыми чувствительными оказались сцинтиграфия с тектротидом с однофотонной эмиссионной компьютерной томографией, совмещенной с компьютерной томографией (ОФЭКТ/КТ) — 71,4% (20/28) и магнитно-резонансной томографией (МРТ) — 90% (18/20). В 35% случаев опухоль была локализована в мягких тканях и в 65% — в костной ткани; при этом наиболее часто опухоль выявлялась в нижних конечностях, далее по частоте следовала локализация в голове. У 18 из 40 пациентов в настоящее время отсутствует ремиссия, и они получают консервативное лечение (препараты фосфора и альфакальцидол (n=15) и буросумаб (n=3)). В случае достижения ремиссии (n=21) наблюдались регресс клинической симптоматики и восстановление костной и мышечной массы. Широкое иссечение опухоли без предварительной биопсии приводило к наилучшему проценту ремиссии — 87%.

**Заключение:**

Заключение. Опухоль-индуцированная остеомаляция наиболее часто встречается у лиц средней возрастной группы, характеризуется тяжелым поражением костной и мышечной ткани с развитием множественных переломов, мышечной слабости и выраженного болевого синдрома. При лабораторной диагностике следует обращать внимание на гипофосфатемию, снижение индекса реабсорбции фосфатов и повышенную активность щелочной фосфатазы. Применение методов функциональной диагностики с препаратами, связывающимися с рецепторами соматостатина 2A подтипа, и МРТ с контрастным усилением являются наиболее точными методами топической диагностики. В случае локализации опухоли рекомендуется широкое иссечение без предварительной биопсии.

## Abstract (en)

**Introduction:**

Introduction: Tumor-induced osteomalacia is an acquired rare disease manifested by hypophosphatemic osteomalacia due to excessive secretion of fibroblast growth factor 23 (FGF23). FGF 23 is a non-classical hormone secreted by bone tissue (osteocytes) and regulates phosphorus metabolism.

The aim of this work is to present clinical experience in the diagnosis, treatment and rehabilitation of patients with tumor-induced osteomalacia.

**Materials and methods:**

Materials and methods: 40 patients with clinically-confirmed tumor-induced osteomalacia were included in the study, 34 of whom had the tumor localized, 27 underwent surgical treatment and 21 achieved stable remission.

**Results:**

Results: The median age was 48 [41; 63] years, 43% were men, the time left from the the onset of the disease was 8 [4; 10] years. Biochemical findings were hypophosphatemia 0.47 [0.4; 0.53] mmol/l, a decrease in the tubular reabsorption phosphate 62 [52; 67]%, and an increase in alkaline phosphatase of 183 [112; 294] units/l. At the time of diagnosis, 100% had multiple pathological fractures, only 10% could move independently, and 77.5% classified the pain as unbearable (8–10 points according to the 10-point pain syndrome scale ). Among the methods used to detect tumors, the most sensitive were scintigraphy with tectrotide with SPECT/CT 71.4% (20/28) and MRI 90% (18/20). In 35% of cases, the tumor was localized in soft tissues and in 65% in bone tissue; The tumor was most often detected in the lower extremities, followed by the head in frequency of localization. 18 patients currently have no remission and they receive conservative treatment (phosphorus and alfacalcidol n=15 and burosumab n=3). In case of achieving remission (n=21), regression of clinical symptoms and restoration of bone and muscle mass was observed. Extensive excision of the tumor without prior biopsy resulted in the best percentage of remission — 87%.

**Conclusion:**

Conclusion: Tumor-induced osteomalacia is characterized by severe damage to bone and muscle tissue with the development of multiple fractures, muscle weakness and severe pain syndrome. In laboratory diagnostics, attention should be paid to hypophosphatemia, a decrease in the tubular reabsorption phosphate index and increased alkaline phosphatase. The use of functional diagnostic methods with a labeled somatostatin analogue to the subtype 2 receptor and MRI with contrast enhancement are the most accurate methods of topical diagnostics. In case of localization of the tumor, a wide excision without a preliminary biopsy is recommended.

## ВВЕДЕНИЕ

Опухоль-индуцированная остеомаляция — редкое приобретенное заболевание, проявляющееся гипофосфатемией и связанными с ней осложнениями. Причиной является фосфатурическая мезенхимальная опухоль (ФМО), которая секретирует фактор роста фибробластов 23 (ФРФ23) [1]. ФРФ23 обладает эндокринными свойствами и приводит к потере фосфатов через почки, а также снижению выработки и усилению катаболизма 1,25-дигидроксивитамина D [2]. У здорового человека способностью секретировать ФРФ23 обладают остеоциты. Опухоли гетерогенны, но их объединяют мезенхимальное происхождение, а также способность секретировать ФРФ23 [3–5]. Гиперсекреция ФРФ23 описана для ряда доброкачественных (атерома, гемангиоперицитома и др.) [6–10] и, реже, злокачественных (рак предстательной железы, рак молочной железы, анапластический рак щитовидной железы, рак толстой кишки, мелкоклеточный рак легкого) опухолей [1][11–18].

Распространенность из-за редкости случаев изучена недостаточно. В 2022 г. вышел метаанализ, объединяющий клиническое описание 1725 случаев со всего мира [19], согласно которому у мужчин заболевание регистрировалось чаще и протекало с большим количеством переломов, чем у женщин. Заболеваемость опухоль-индуцированной остеомаляцией была оценена в 2015 г. в Японии [20] и составила 0,04:100 000 человек в год, что намного реже, чем заболеваемость схожим по клинической картине Х-сцепленным гипофосфатным рахитом (XLH), которая составляет 5:100 000 новорожденных [21]. Необходимо отметить спорадичность проспективных исследований ФРФ23-секретирующих опухолей в мировой практике и небольшие размеры выборок, что подчеркивает значимость этой работы [22–28]. Опухолевая остеомаляция встречается, как правило, во взрослом возрасте и требует тщательной дифференциальной диагностики.

Заболевание протекает тяжело за счет гиперсекреции ФРФ23. Возможно метастазирование в случае повреждения целостности образования. У пациентов наблюдаются выраженные боли в костях (до 99,3%), множественные переломы (до 79%), уменьшение в росте (до 69%), генерализованная миопатия (до 65%), а также ряд неспецифических симптомов [1–3][22][23]. Заболевание диагностируется в среднем спустя 4–8 лет после начала симптомов. Это связано с несколькими факторами. Во-первых, редкое определение уровня фосфора в крови. Во-вторых, во всем мире отмечается низкая осведомленность врачей о ФРФ23-опухолях. В-третьих, неспецифичность симптомов усложняет диагностику. В-четвертых, измерение ФРФ23 в сыворотке доступно лишь в ограниченном числе стран [3][24][25].

Помимо поздней диагностики, остается значительное количество нерешенных клинических проблем, связанных с трудностями локализации образований и отсутствием клинических рекомендаций по лечению.

Локализация опухолей, продуцирующих ФРФ23, строится поэтапно и включает функциональную, анатомическую визуализацию и, при возможности, селективный забор крови с определением концентраций ФРФ23. Для функциональной визуализации применяют методы, основанные на сродстве диагностических радиофармпрепаратов (РФП) к опухолевым рецепторам (соматостатиновые 2А типа SSTR2A): сцинтиграфия с РФП (99mTc-тектротид, 111In-октреотид) либо позитронно-эмиссионная томография (ПЭТ, ПЭТ/КТ) с РФП (соли галия: Ga DOTATATE/DOTATOC/DOTANOC) [26][27]. Широко используемый препарат для поиска новообразований — 18F-фтордезоксиглюкоза (18F-ФДГ) — не показал успешных результатов в диагностике ФРФ23-опухолей. Использование 18F-ФДГ обладает меньшей чувствительностью по сравнению со сцинтиграфией, а лучшим визуализирующим методом считается ПЭТ/КТ с Ga DOTATATE [26][27]. Также отметим, что 21% опухолей не имеют на своей поверхности рецепторов SSTR2A, а значит, их поиск на данный момент затруднен [1][2]. Для анатомической визуализации, в зависимости от расположения и природы опухоли, применяют магнитно-резонансную томографию (МРТ), мультиспиральную компьютерную томографию (МСКТ), ультразвуковое исследование (УЗИ). Селективный забор крови с определением концентраций ФРФ23 был предложен как вариант диагностики в сомнительных случаях и уже показал свою эффективность [28][29].

При поэтапном подходе удается диагностировать около 70% опухолей [1, 2], а комбинация различных методов визуализации с селективным забором крови, вероятно, могла бы повысить чувствительность. Из 1725 описанных в мире случаев опухоли были выявлены у 1493 (87%) пациентов [19]. Образования обнаруживаются в мягких тканях (55%) или в костях (45%) любой локализации. Чаще поражаются нижние конечности, голова и суставы. Размеры небольшие и составляют 1–2 см [30].

Полное излечение пациента возможно в случае радикального удаления опухоли, в отличие от врожденных форм остеомаляции [30][31]. Отсутствие ремиссии наблюдается при невозможности удалить патологический очаг, а рецидивы встречаются при нерадикальном хирургическом лечении и малигнизации образования. В таких случаях показана медикаментозная терапия.

Прогноз после постановки диагноза в основном зависит от локализации опухоли, степени злокачественности и стратегии лечения [1][32]. Консервативная терапия (активные и нативные формы витамина D, а по показаниям препараты фосфора) назначается, если опухоль неоперабельна или не локализована, а также при подготовке к оперативному лечению. Однако эти лекарства могут приводить к вторичному и третичному гиперпаратиреозу, а затем к хронической почечной недостаточности [33]. За рубежом разработан и применяется препарат Буросумаб, являющийся человеческим моноклональным антителом к ФРФ23.

## ЦЕЛЬ

Целью настоящей работы стал анализ клинических проявлений, лабораторных изменений и диагностических возможностей методов локализации ФМО.

## МАТЕРИАЛЫ И МЕТОДЫ

## Место и время проведения исследования

Проведено несравнительное проспективное одноцентровое исследование пациентов, обратившихся за медицинской помощью с 2016 по 2022 гг. Источниками информации являлись данные обследования и анкетирования пациентов, а также медицинская документация (истории болезней и амбулаторные карты).

Критерии включения: мужской и женский пол, возраст более 18 лет, клинический диагноз «Фосфопеническая форма остеомаляции опухолевого генеза» (Е83.3 Нарушения обмена фосфора или M83.8 Другая остеомаляция у взрослых). Диагноз устанавливался при совокупности типичных клинических, лабораторных и инструментальных признаков ФРФ23-продуцирующей опухоли [1][34], при исключении иных причин потери фосфатов.

Критерии исключения: гипофосфатемическая остеомаляция, вызванная другими причинами. Исключались пациенты с острыми состояниями (алкалоз, ацидоз, передозировка инсулином, декомпенсированный сахарный диабет, синдром «голодных костей», рефидинг-синдром, цирроз, острая почечная и печеночная недостаточность); потерями фосфора с мочой (синдром Фанкони, первичный гиперпаратиреоз); нарушениями абсорбции фосфора в ЖКТ (алкоголизм, мальабсорбция, прием антацидов, дефицит питания); выраженным дефицитом витамина D; лекарственной гипофосфатемией, вызванной внутривенным введением препаратов железа; наследственными рахитами; заболеваниями крови.

Собиралась информация и оценивались следующие признаки.

Анализ данных производился в пакете статистических программ IBM SPSS Statistics Base (SPSS, США). Описательная статистика: распределение количественных признаков считалось нормальным, если по результатам расчета критериев Колмогорова–Смирнова и Шапиро–Уилка отмечались значения p>0,05. Поскольку количественные признаки были распределены ненормально, то для описательной статистики данные представлены в виде медианы и 1-го, 3-го квартилей. Качественные параметры представлены в процентах. Для оценки изменений количественных параметров в независимых выборках, имеющих неправильное распределение переменных, использовался критерий Манна–Уитни. Различия между двумя группами, имеющими нормальное распределение, сравнивались с помощью t-критерия Стьюдента. Для сравнения параметров в зависимых выборках, не подчиняющихся закону нормального распределения, использовался критерий Вилкоксона. Различия между двумя или тремя группами сравнивались с помощью точного критерия Фишера. Различия показателей считались статистически значимыми при уровне значимости (p) меньше 0,05. Исследование было одобрено локальным этическим комитетом.

## РЕЗУЛЬТАТЫ

За период наблюдения диагноз фосфопенической остеомаляции верифицирован у 40 пациентов. Опухоль выявлена у 34 человек (85%), и 27 (79%) подверглись хирургическому лечению, а 21 (78%) из них достигли ремиссии (рис. 1).

**Figure:**
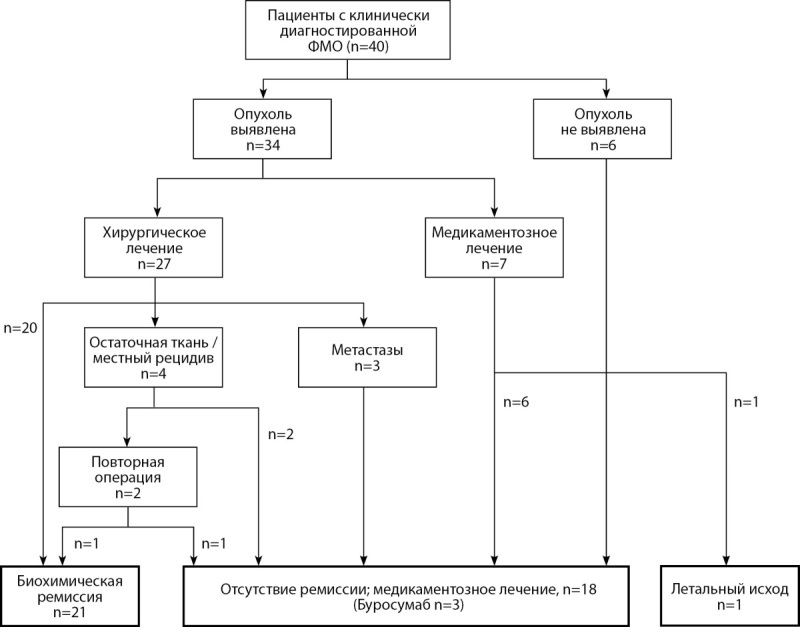
Рисунок 1. Распределение пациентов в ходе исследования. Наблюдались 40 пациентов, и 21 из них достигли ремиссии.

За время исследования зарегистрирован 1 случай смерти, когда пациентка скончалась от дыхательной недостаточности ввиду выраженной деформации грудной клетки, и 1 случай жизнеугрожающего гемолиза эритроцитов с уровнем гемоглобина 37 г/л, потребовавший срочного переливания крови. На момент последнего визита 18 пациентов (45%) лечились консервативно, в том числе 3 пациента получали Буросумаб.

## Общие характеристики пациентов

Возраст (Me [ Q1; Q3]) на момент обследования составил 48 [ 41; 63] лет (мин. 19; макс. 73 года). Семнадцать пациентов (43%) — мужчины. Время от появления симптомов до установления диагноза (Me [ Q1; Q3]) составило 8 [ 4; 10] (мин. 2; макс. 24 года) (табл. 1). Кроме того, зафиксированы осложнения, связанные с избытком ФРФ23: вторичный гиперпаратиреоз — n=13 (33%), нефролитиаз — n=11 (28%), анемия — n=9 (23%).

**Table:** Таблица 1. Клинико-возрастные характеристики пациентов Примечание. Данные представлены в виде Me [ Q1; Q3].

Показатель	Все пациенты (n=40)	Мужчины (n=17)	Женщины (n=23)
Возраст, лет	48 [ 41; 63]	47 [ 43; 58]	50 [ 41; 65]
Рост, см	157 [ 148; 166]	173 [ 165; 178]	150 [ 147; 154]
Вес, кг	77 [ 51; 84]	82 [ 78; 98]	60 [ 48; 77]
ИМТ, кг/м²	28 [ 22; 33]	28 [ 25; 30]	24 [ 22; 33]
Длительность заболевания, лет	8 [ 4; 10]	8 [ 4; 10]	8 [ 3,5; 10]

## Качество жизни

Согласно шкале EQ-5D, был рассчитан индекс EQ-5D (табл. 2). Исходно медиана индекса составила -0,074, а спустя 12 мес ремиссии — 0,624 (p=0,005). Медиана Δ индекса EQ-5D составила 0,55 [ 0,49; 0,82], что означает выраженный эффект. Интерпретация эффективности лечения по индексу EQ-5D проводилась в соответствии с его градацией: минимальное клинически значимое изменение индекса EQ-5D соответствовало разнице показателей до и после лечения, равной 0,10. ΔEQ-5D<0,10 означало отсутствие эффекта; 0,10≤ΔEQ-5D≤0,24 — минимальный эффект; 0,24≤ΔEQ-5D<0,31 — удовлетворительный эффект; ΔEQ-5D≥0,31 — выраженный эффект [35–37].

**Table:** Таблица 2. Динамика значений опросника EQ-5D у пациентов с ФРФ23-опухолью *Оценивались 5 разделов опросника EQ-5D. Опросник описывает проблемы, связанные с D1 — перемещением индивидуума в пространстве, D2 — уходом за собой, D3 — привычной повседневной деятельностью, D4 — определением наличия боли или дискомфорта, D5 — наличием депрессии. Каждый раздел оценивался в зависимости от степени выраженности проблемы: 1 — нет нарушений, 2 — есть умеренные нарушения, 3 — есть выраженные нарушения. В результате каждый пациент получал свой профиль качества жизни, например: 22331. Теоретически было возможно получение 245 вариантов состояний здоровья. Далее определяется индивидуальный EQ-5D-индекс — согласно таблице, составленной по результатам опроса репрезентативной выборки европейской и азиатской популяции. EQ-5D-индекс определяется от состояния полного здоровья, принятого за «1», до состояния смерти, принятого за «0». Однако некоторые состояния здоровья оценивались респондентами «хуже смерти», поэтому имели отрицательные значения [36].

Группа больных	Число обследованных больных	EQ-5D-индекс*, Me [ Q1; Q3]	P-value
До лечения	29	-0,074 [ -0,074; 0,081]	0,005
Ремиссия 12 мес	13	0,624 [ 0,48; 0,779]

Аналогичная динамика отмечалась и по другим критериям, в частности по возможности передвигаться самостоятельно (рис. 2): исходно лишь 10% (4 из 40) могли передвигаться самостоятельно без помощи средств опоры, а после достижения ремиссии — 76% (p<0,001).

Кроме того, пациентам предлагалась визуальная аналоговая 10-балльная шкала боли. Оценивая интенсивность боли в течение настоящего заболевания, подавляющее большинство исследуемых называли высокие дескрипторы боли: 93% (38 из 40) указывали на интенсивность боли 5 баллов и выше, а 77,5% (31 из 40) — выше 8 баллов (рис. 3). После достижения ремиссии у 100% обследуемых боль не превышала 5 баллов, а 61,9% говорили об отсутствии боли (1–2 балла) (p<0,001).

**Figure:**
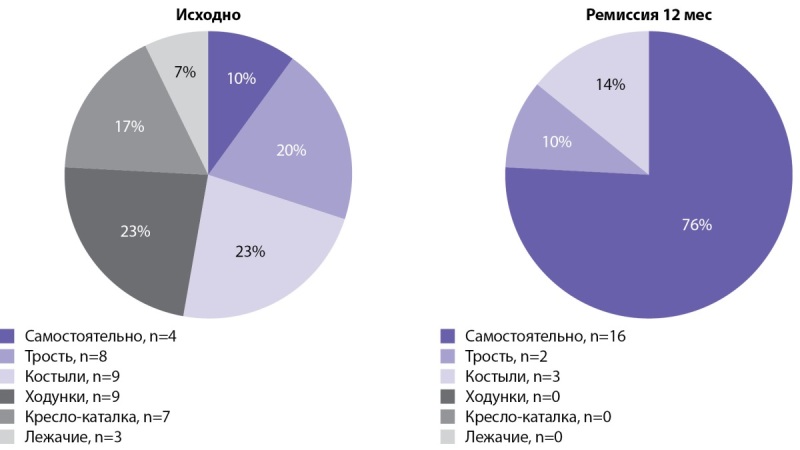
Рисунок 2. Использование вспомогательных средств для передвижения исходно (n=40) и в период ремиссии (n=21).

**Figure:**
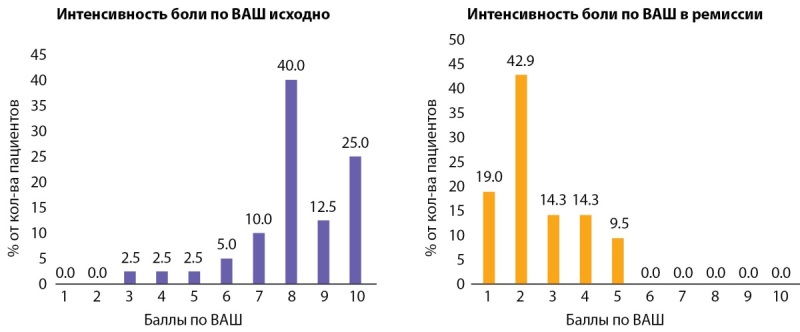
Рисунок 3. Интенсивность боли по ВАШ исходно (n=40) и в период ремиссии (n=21).

## Лабораторные тесты

Лабораторно исходно определялись гипофосфатемия, низкий индекс тубулярной реабсорбции фосфатов, высокая активность щелочной фосфатазы и низко-нормальные концентрации 25-гидроксивитамина D. Уровни кальция, креатинина, остеокальцина, С-концевого телопептида (Ctx) находились в пределах референсных интервалов (табл. 3).

У 13 (33%) пациентов наблюдалось повышение концентраций ПТГ, на фоне нормокальциемии — n=7 (53,8%) и гиперкальциемии — n=6 (46,2%).

**Table:** Таблица 3. Лабораторные характеристики пациентов Примечание. Данные представлены в виде Me [ Q1; Q3].

Показатель	Все пациенты (n=40)	Мужчины (n=17)	Женщины (n=23 )	P-value
Фосфор сыворотки, ммоль/л (0,74–1,52)	0,47 [ 0,4; 0,53]	0,47 [ 0,39; 0,49]	0,49 [ 0,4; 0,58]	0,14
Общий кальций сыворотки, ммоль/л (2,15–2,55)	2,33 [ 2,21; 2,48]	2,35 [ 2,26; 2,42]	2,3 [ 2,19; 2,53]	0,6
Креатинин сыворотки, мкмоль/л (50–98)	64,8 [ 58; 72,3]	71,8 [ 65,2; 83,1]	62 [ 54; 67]	0,25
25-гидроксивитамин D, нг/мл ( >30)	28 [ 18,5; 36]	29 [ 24,8; 41,5]	27 [ 18; 34]	0,9
С-концевой телопептид, нг/мл	0,6 [ 0,39; 0,93]	0,6 [ 0,46; 0,99]	0,48 [ 0,35; 0,88]	0,834
Остеокальцин, нг/мл (15–46)	24 [ 16,74; 40,18]	24,76 [ 20; 33,61]	23,23 [ 14,61; 52,2]	0,25
ПТГ, пг/мл (15–65)	59 [ 36; 114]	46,46 [ 31,98; 73]	81 [ 43,98; 153]	0,6
Щелочная фосфатаза, Ед/л (40–150)	183 [ 112; 294]	196 [ 147,5; 280,5]	156 [ 99; 267,5]	0,4
Фосфор разовой мочи, ммоль/л (12,9–43,9)	19,47 [ 13,15; 33]	26,5 [ 21,95; 36,84]	14,77 [ 12,44; 30]	0,75
СКФ (EPI) (>60 мл/мин/1,73 м²)	102 [ 86; 112]	107 [ 90; 112]	102 [ 84; 112]	0,11
Индекс реабсорбции фосфатов, % (85–95%)	59 [ 50,17; 72,25]	62 [ 52; 67]	58 [ 48,45; 72,5]	0,75

## Визуализирующие тесты

У пациентов проводился поиск опухолей. Соблюдалась этапность, т.е. сначала проводилась функциональная визуализация. Либо с помощью сцинтиграфии всего тела с меченными технецием-99m аналогами соматостатина (99mTc-тектротид — чувствительность к SSTR2а/b и SSTR5, в меньшей мере к SSTR3), дополненной ОФЭКТ/КТ зоны интереса, либо с помощью ПЭТ/КТ с РФП, меченными галлием-68: DOTА-ТАTE (чувствительность к SSTR2a и SSTR2b) и DOTA-NOC (чувствительность к SSTR3 и SSTR5). На следующем этапе, после обнаружения SSTR-позитивного очага, проводилась анатомическая визуализация методами МРТ и МСКТ с внутривенным контрастированием. Чувствительность методов функциональной визуализации составила 53,8% (7/13) для ПЭТ/КТ, 71,4 % (20/28) для сцинтиграфии всего тела с ОФЭКТ-КТ. Среди методов второго этапа диагностики наибольшей чувствительностью обладала МРТ 90% (18/20), а для МСКТ — это 63% (17/27).

## Размер и расположение новообразований

Опухоль найдена у 85% (34/40) пациентов (рис. 4). Из них в мягких тканях располагалось 35% опухолей (12/34), а в костях — 65% (22/34). Размер опухолей до 10 мм обнаружен у 20% (7/34), 10–20 мм — у 56% (19/34), а более 20 мм — у 24% (8/34) пациентов.

**Figure:**
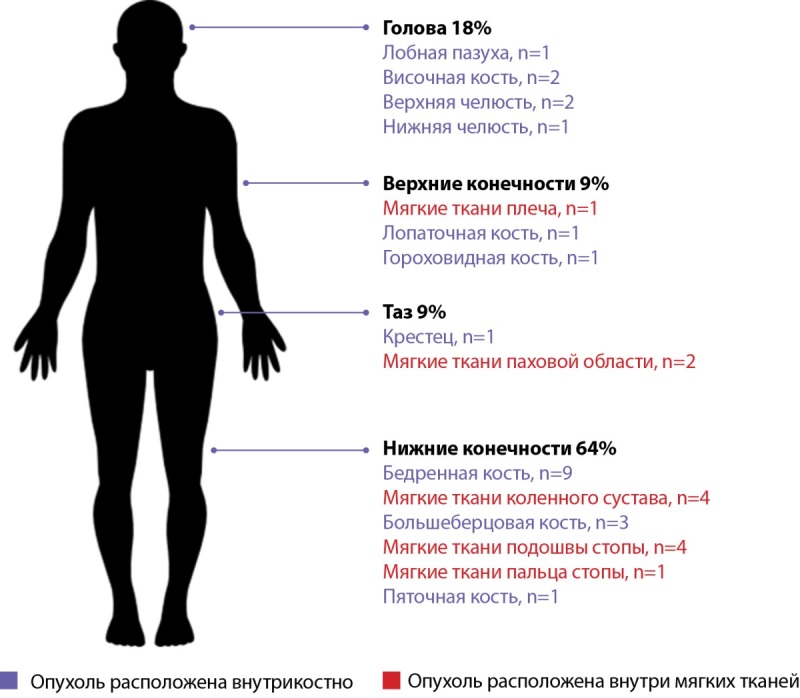
Рисунок 4. Локализация ФРФ23-образований.

## Хирургическое лечение

Среди 27 пациентов, перенесших операцию, 20 человек (74%) достигли биохимической ремиссии после первой операции (см. рис. 1). Одна пациентка повторно прооперирована по поводу остаточной ткани через 1 нед после 1-й операции, что привело к общей частоте ремиссии у 21 человек (78%). У 3 (11%) пациентов были обнаружены отдаленные метастазы после первой операции. Отметим, что именно эти пациенты подвергались предоперационной биопсии. У оставшихся 3 человек (11%) обнаружены местные рецидивы, видимо, ввиду нерадикально проведенного хирургического вмешательства.

При анализе исходов в зависимости от объема хирургического вмешательства (рис. 5) частота ремиссии была самой высокой у тех, кто получил широкое иссечение (20/23, 87%). Резекция по краю опухоли (1/2, 50%), проведенная двум пациентам, показала сомнительные результаты, т.к. у обоих после первой операции не было ремиссии, но повторная операция привела к ремиссии у одного пациента. Выскабливание, по нашим данным, к ремиссии не привело (0/2, 0%).

Если опухоль обнаруживалась в конечностях, то частота наступления ремиссии была выше — 64% для нижних конечностей и 67% для верхних, что, по-видимому, связано с большими возможностями для широкого иссечения. Опухоли, располагающиеся в голове и в области таза, показали меньший процент ремиссии — 50 и 33% соответственно.

Семь пациентов с обнаруженными опухолями не подверглись хирургическому вмешательству из-за метастазов (n=1), предпочтения пациента (n=1), потенциальных хирургических осложнений (n=4), смерти пациента (n=1).

**Figure:**
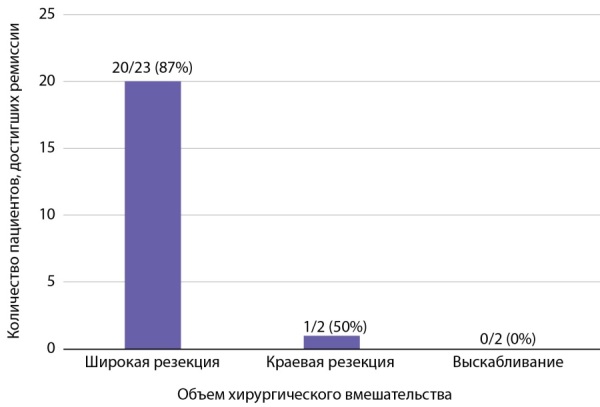
Рисунок 5. Частота наступления ремиссии в зависимости от объема операции.

## Консервативная терапия

Все пациенты получали препараты альфакальцидола, медиана доз 3,5 [ 3; 4] мкг/сут (мин. 1, макс. 8), и колекальциферола — 2000 [ 2000; 2000] МЕ/сут. Чтобы поддержать уровень фосфора в нижне-нормальном диапазоне, препараты фосфора потребовались 29 (73%) пациентам в дозе 1,44 [ 1,44; 1,65] г/сут (мин. 1,2, макс. 2,5). Такая терапия позволила достичь снижения симптомов у большинства пациентов (n=28), однако биохимической ремиссии — лишь у малой части (n=8 (20%)).

Трем пациентам был назначен Буросумаб, поскольку терапия препаратами альфакальцидола и фосфора не привела к улучшению состояния (n=3). Возраст пациентов, получающих Буросумаб, составил 53, 48 и 45 лет, 2 мужчин и 1 женщина. Самый длительный период приема препарата составил 1 год в дозе 0,6 мг/кг в месяц, 2-й пациент принимает препарат 6 мес в дозе 0,5 мг/кг в месяц, 3-й пациент — 4 мес в дозе 1,8 мг/кг в месяц в настоящее время. На фоне Буросумаба, согласно инструкции, отменена терапия препаратами альфакальцидола и фосфора и удалось достичь биохимической ремиссии и выраженного снижения симптомов у всех 3 пациентов. Серьезных нежелательных явлений не зафиксировано.

## Костная система

Исходно переломы и снижение роста за жизнь диагностированы у 40 (100%) пациентов. Псевдопереломы выявлены у 15 (38%). Медиана количества переломов у одного человека составила 14 [ 10; 20]. Их локализация и частота представлены на рисунке 6. Чаще всего фиксировались переломы позвонков, ребер, шейки бедренной кости, костей таза. В случае достижения биохимической ремиссии в течение 12 мес новые переломы не произошли ни у одного пациента.

**Figure:**
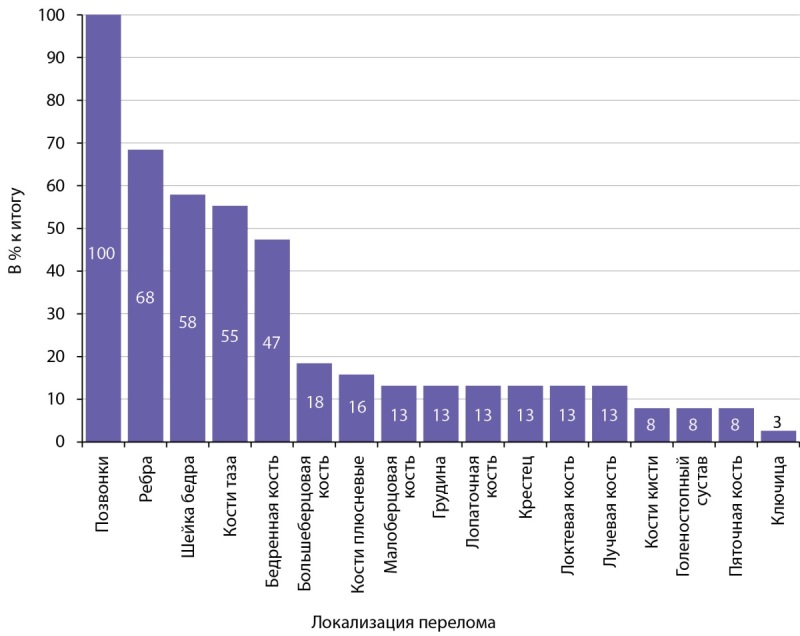
Рисунок 6. Локализация переломов у пациентов с ФРФ23-опухолью.

По данным рентгеновской денситометрии пациентов старше 50 лет исходно не выявлено выраженного снижения минеральной плотности кости (МПК): медиана по T-критерию составила в позвоночнике -0,9, в шейке бедренной кости -2,1. Тем не менее после наступления ремиссии выявлен прирост МПК: в позвоночнике медиана прироста составила 1,9, в шейке бедренной кости — 2,2, что статистически значимо по обеим локализациям (табл. 4).

Кроме того, исходно медиана трабекулярного костного индекса составила 1,285 и была ниже нормы (1,310), а в период ремиссии отмечена нормализация показателя, однако статистически незначимая (табл. 4).

**Table:** Таблица 4. Данные рентгеновской денситометрии

Показатель	Исходно Me [ Q1; Q3]	Ремиссия Me [ Q1; Q3]	P-value
МПК L1–L4, T-score (n=16)	-0,9 [ -2,2; 0]	1,0 [ 0,6; 1,7]	0,046
МПК Ferum Neck, T-score (n=16)	-2,1 [ -3,4; -1,25]	0,1 [ -1,2; 1,3]	0,043
Трабекулярный костный индекс, TBS (n=16) >1,310	1,285 [ 1,156; 1,405]	1,374 [ 1,283; 1,577]	0,225

## Мышечная система

Снижение мышечной силы (предполагаемой саркопении) по данным кистевой динамометрии исходно выявлено у 10 из 17 пациентов. Снижение массы мышц (т.е. наличие саркопении) по данным индекса скелетных мышц (ASMI или ИАСМ) исходно отмечалось у 1 из 10 дообследованных пациентов. Отмечено увеличение ASMI спустя 12 мес ремиссии: медиана у мужчин возросла с 9,24 [ 8,3; 9,74] до 11,48 [ 11,45; 11,51] кг/м² , а у женщин — с 6,99 [ 6,96; 7,65] до 8,13 [ 7,5; 8,3] кг/м². Судить о статистической достоверности не представляется возможным из-за малого размера выборки. Низкая работоспособность скелетной мускулатуры по результатам NIH Toolbox 4 Meter Walk Gait Speed Test исходно выявлена у 20 из 22 пациентов.

## ОБСУЖДЕНИЕ

В этом исследовании мы обобщили клинические, лабораторные и инструментальные данные с целью представить мультидисциплинарный взгляд на пациентов с ФРФ23-продуцирующими опухолями. Чаще заболевание встречается у лиц среднего возраста, в равной степени у мужчин и женщин. Диагноз, согласно данному исследованию, верифицируется спустя 8 лет после появления симптомов, что является большим сроком, и у пациентов успевают развиться множественные переломы. В Китае и Японии диагноз верифицируется быстрее, а именно спустя 2,9 и 2,4 года соответственно [3][25]. Поздняя диагностика, по-видимому, связана с рядом причин. Во-первых, это низкая осведомленность врачей, т.к. заболевание редко встречается. Согласно японским данным, пациенты обращаются к ортопедам (56/88, 63,6%), врачам общей практики (12/88, 13,6%), эндокринологам (3/88, 3,4%), ревматологам (2/88, 2,3%) и неврологам (6/88, 6,8%), а значит, именно их нужно информировать в первую очередь [25]. Во-вторых, неспецифичностью скелетно-мышечных симптомов. В-третьих, отсутствием скрининга фосфора крови и его поздним определением у людей с нарушениями опорно-двигательного аппарата. Наконец, с недоступностью в России определения ФРФ23, в отличие от Японии, где это возможно с 2019 г.

Поздняя диагностика и прогрессирование заболевания приводят к стремительному снижению качества жизни пациентов, что проявляется выраженным болевым синдромом, неспособностью самостоятельно передвигаться и заниматься повседневной деятельностью, а также нарастают тревожность и депрессия. Все в совокупности ухудшает ситуацию и делает течение заболевания изнуряющим. С другой стороны, в случае успешного лечения болевой синдром исчезает, физические возможности расширяются и качество жизни повышается.

Лабораторные признаки в когорте исследуемых соответствовали ФРФ23-зависимым нарушениям. В случае успешного хирургического лечения уровень фосфора восстанавливался в течение 5–14 дней после операции. В послеоперационном периоде возможен подъем уровня фосфора, что, вероятно, связано с компенсаторными механизмами. В течение года показатели, как правило, нормализовались. Интересно отметить, что у 33% отмечалось повышение уровня ПТГ. Поскольку гипофосфатемия, а также нарушения скелетно-мышечного аппарата встречаются как при первичном гиперпаратиреозе, так и при избытке ФРФ23, то возникает проблема дифференциальной диагностики. Избыток ФРФ23 влечет за собой дефицит активного метаболита витамина D (D-гормона, или 1,25(ОH)D3), что может способствовать развитию вторичного гиперпаратиреоза, а также кальций-чувствительные рецепторы перекрестно реагируют на гипофосфатемию [38], что, в свою очередь, может приводить к повышению ПТГ с гиперплазией околощитовидных желез и даже развитием третичного гиперпаратиреоза. По нашему опыту, в таких случаях наилучшим методом дифференциальной диагностики могло бы служить определение в крови ФРФ23. Если это недоступно, тогда мы предлагаем ориентироваться на следующие параметры. Во-первых, на нормокальциемию, которая более характерна для ФРФ23-зависимых нарушений, во-вторых, на степень гипофосфатемии, которая чаще более выражена при ФРФ23-зависимых нарушениях. В-третьих, на концентрацию паратгормона, который выше при гиперпаратиреозе.

Различные комбинации визуализирующих тестов позволили найти опухоль у 34/40 пациентов (85%). Не описаны специфические лучевые признаки, и опухоль легко «пропустить», если не соблюдать поэтапный подход: вначале функциональная визуализация, затем анатомическая. Для ФМО характерно наличие единичного SSTR2A-позитивного очага. Если визуализируется два очага и более, то это должно настораживать и может свидетельствовать либо о метастазах (первично не характерны, встречаются после нерадикальной операции либо после биопсии), о злокачественной природе новообразования (встречается очень редко), либо об ошибочном диагнозе и требует дифференциального поиска с неспецифическим разрушением костей (переломами, бурыми опухолями, остеомаляцией, фиброзным остеитом и др.).

Мы соблюдали этапный подход, т.е. МРТ и МСКТ проводились после функциональной визуализации с прицелом на выявленный SSTR2A-позитивный очаг. По нашим данным, наиболее чувствительными методами оказались сцинтиграфия с 99mTc-тектротидом (71,4% (20/28)) и МРТ (90% (18/20)). В зарубежных работах лидерами являются ПЭТ-КТ с 68GaDOTATATE и сцинтиграфия с 99mTc-тектротидом, 111In-октреотидом [26][27]. Согласно японскому наблюдению 30 пациентов, комбинация двух визуализирующих тестов (ПЭТ/КТ+сцинтиграфия) обладала большей эффективностью (89%) в сравнении с одним методом (72%). Если дополнительно проводился селективный забор крови, то образования обнаруживались в 100% случаев [28][29]. Высокопольная МРТ и МСКТ позволяют уточнить наличие капсулы или остеосклероза. Их отсутствие предполагает инфильтративный рост опухоли и требует выполнения операции с соблюдением онкологических принципов радикальности и абластичности. Размеры и расположение образований соответствуют зарубежному опыту, т.е. обычно опухоли малых размеров: в 76% (26/34) до 2 см. Располагаются чаще внутрикостно либо в мягких тканях. Природа опухоли остается предметом изучения. Опухоли гетерогенны, имеют мезенхимальную природу. Поэтому в литературе описаны случаи обнаружения ФРФ23-опухолей не только в костях и в мышцах, но и в жировой ткани [10], в околоушной железе [8].

Хирургическое лечение — это метод выбора, и первая операция является главным шансом излечить пациента. Операция должна проводиться в специализированном отделении онкохирургом высокой квалификации. Исход хирургического лечения во многом зависит от локализации образования. Рекомендуется широкая резекция, т.к. это приводит к наивысшему проценту ремиссии: у нас это 87% (20/23), в Японии (ретроспективное наблюдение) — 93,8% (15/16) [25]. Если широкая резекция невозможна, например, из-за локализации опухоли, то обсуждается эффективность краевой резекции. Так, в нашем исследовании ремиссии достиг 1 из 2 прооперированных пациентов, в Японии — 65,2% (15/23). Вариант выскабливания сомнителен, по нашим данным, выскабливание к ремиссии не привело — 0% (0/2). Выскабливание опухоли было выполнено для образований, располагающихся в костях черепа, где невозможно было выполнить широкую резекцию. Результаты демонстрируют, что опухолевые клетки, оставленные в ране, способны секретировать ФРФ23 и приводить к гипофосфатемии. Отмечено, что нерадикальное удаление ФРФ23-опухоли ведет не только к повторному прогрессирующему снижению уровня фосфора крови и нарастанию проявлений остеомаляции, но и к прогрессированию опухолевого процесса [32]. С другой стороны, краевая резекция, по данным ретроспективного японского наблюдения, в малом проценте случаев была эффективна. Возможно, это связано с характером роста опухоли. Гистологически описано, что опухоли гетерогенны и различаются по строению; чаще имеют инфильтративный характер роста, однако встречаются образования с капсулой без инфильтрации окружающих тканей [39]. Согласно нашему и зарубежному опыту, мы не рекомендуем проводить предоперационную биопсию, поскольку она ассоциирована с отдаленными метастазами. Как альтернативный метод за рубежом предлагают проводить селективный забор крови из вен, оттекающих от опухоли и от симметричного участка с определением концентраций ФРФ23 [28][29].

Остеомаляция, индуцированная опухолью, в редких случаях может быть связана со злокачественными новообразованиями, такими как рак предстательной железы [12][13], рак молочной железы [14], анапластический рак щитовидной железы [15], рак толстой кишки [16], мелкоклеточный рак легкого [17], все из которых являются немезенхимальными опухолями, но претерпевают эпителиально-мезенхимальный переход в процессе метастазирования. В нашем исследовании зафиксирован 1 случай рака предстательной железы с секрецией ФРФ23 у мужчины 69 лет [18]. В литературе описано, что злокачественные новообразования с продукцией ФРФ23 растут и метастазируют с очень низкой скоростью, и пациенты выживали в течение многих лет [25]. В нашем исследовании после назначения традиционной, консервативной терапии пациент отметил улучшение общего состояния, возобновил самостоятельное передвижение. Обсуждается назначение препаратов — блокаторов рецепторов ФРФ23 (FGFR1–4), однако из-за широкого распространения рецепторов в организме наблюдается выраженный токсический эффект, и терапия должна быть дополнительно изучена и ограничена пациентами с метастазами [40].

В описанной нами когорте все пациенты в тот или иной период времени получали консервативное лечение препаратами альфакальцидола и колекальциферола. Практически три четверти пациентов (73%) нуждались в назначении препаратов фосфора. Если сравнивать эффективность традиционной терапии с лечением Буросумабом, то биохимической ремиссии удалось достичь у малой части (n=8/40 (20%)), в отличие от Буросумаба (n=3/3 (100%)). Тем не менее долгосрочные результаты терапии Буросумабом еще неизвестны и требуют изучения. Есть отдельные публикации о попытках лечения аналогами соматостатина с радиоактивными метками и о проведении радиочастотной абляции, но они не продемонстрировали должного эффекта и требуют дополнительных исследований [19][41]. В данной работе 1 пациент с отдаленными метастазами ФРФ23-опухоли получал в течение 6 мес лечение аналогами соматостатина без радиоактивных меток, но без эффекта.

В обследуемой когорте у большинства пациентов определялись признаки снижения работоспособности и предполагаемой саркопении, т.е. прогрессирующего генерализованного повреждения скелетной мускулатуры [5][42]. Однако одновременное снижение мышечной массы, т.е. наличие тяжелой саркопении, подтверждено у 1 из 10 обследованных пациентов. Данные утверждения требуют дополнительных исследований, поскольку из-за малой численности группы говорить о достоверных изменениях не представляется возможным. Хотя, несомненно, пациенты жалуется именно на «слабость в мышцах», которая исчезает в случае ремиссии. Однако инструментально в данном исследовании удалось показать лишь статистически незначимое увеличение индекса массы мышц (ASMI) в ремиссию. Тем не менее эти данные свидетельствуют о значительном вкладе гипофосфатемии в патогенез саркопении и призывают врачей обращать внимание на состояние мускулатуры. Мышечная масса — самая большая ткань в организме, и она, видимо, подвержена поражению при гипофосфатемии, что приводит к повышению риска падений, переломов, физической нетрудоспособности и увеличению смертности.

Состояние и динамика восстановления костной массы представляют определенный интерес. В разгар заболевания множественные переломы выявлены у 100% пациентов. Наиболее частая локализация — позвонки, а также ребра, шейка бедра и кости таза. Вот почему мы рекомендуем проводить скрининг уровня фосфора всем пациентам с нарушениями минерального обмена. В случае достижения биохимической ремиссии новые переломы не произошли ни у одного пациента.

Интересная работа была проведена в Китае [43], где оценивались показатели денситометрии и трабекулярного костного индекса (Trabecular Bone Score, TBS) у 186 пациентов с клинически подтвержденной опухоль-индуцированной остеомаляцией. Любопытно, что у пациентов чаще встречалось поражение микроархитектоники костной ткани (TBS<1,35), чем снижение МПК (L1–4 Z-score ≤-2): 89,6% vs 43,9%, p<0,001. В нашей работе эти нарушения встречались с одинаковой частотой: 56,25% vs 54,55%

В конце 2022 г. впервые были опубликованы глобальные клинические рекомендации по ведению пациентов с опухоль-индуцированной остеомаляцией [44].

В текущем исследовании были некоторые ограничения. Во-первых, не все пациенты обследовались проспективно, часть данных была взята из медицинских документов. Во-вторых, в это исследование были включены пациенты с клинически диагностированной ФРФ23-опухолью, однако 6 образований (15%) не удалось найти. В Японии не удалось найти 27% опухолей [25]. Отметим, что мы предварительно исключали все известные иные причины фосфопенической остеомаляции, включая инфузии препаратов железа, врожденные рахиты, нарушения всасывания фосфора и т.д.

## ЗАКЛЮЧЕНИЕ

В данном исследовании мы обобщили клинические, лабораторные и инструментальные данные с целью представить мультидисциплинарный взгляд на пациентов с ФРФ23-продуцирующими опухолями. Необходимо улучшить диагностику заболевания путем информирования врачей, определения фосфора в крови у пациентов с различными скелетно-мышечными нарушениями, а также введением в лабораторную практику определения ФРФ23. Несмотря на тяжесть симптомов, более половины пациентов достигают биохимической ремиссии с исчезновением болевого синдрома, повышением качества жизни и восстановлением скелетно-мышечного аппарата. Современные визуализирующие тесты и поэтапный подход позволяют найти опухоль в 85% (34/40) случаев.

В качестве стратегии лечения мы рекомендуем хирургическое лечение с широким иссечением. Предоперационная биопсия не рекомендуется, а в случае наличия метастазов или невозможности удалить образование показана консервативная терапия. Лечение Буросумабом показало хорошие результаты с достижением биохимической ремиссии, и, возможно, длительная терапия сможет улучшить и другие показатели пациента.

## ДОПОЛНИТЕЛЬНАЯ ИНФОРМАЦИЯ

Источники финансирования. Работа проведена при финансовой поддержке Министерства здравоохранения Российской Федерации в рамках выполнения государственного задания «Разработка персонализированных подходов к диагностике и лечению пациентов с остеопорозом вследствие эндокринопатий на основании изучения молекулярно-генетических предикторов, применения инновационных методов диагностики и исследования патогенеза редких заболеваний скелета» № АААА-А20-120011690202-4.

Конфликт интересов. Авторы декларируют отсутствие явных и потенциальных конфликтов интересов, связанных с содержанием настоящей статьи.

Участие авторов. Все авторы внесли существенный вклад в написание рукописи, существенные правки, что повысило научную ценность статьи, и одобрили финальную версию статьи перед публикацией, выразили согласие нести ответственность за все аспекты работы, подразумевающую надлежащее изучение и решение вопросов, связанных с точностью или добросовестностью любой части работы.
